# A longitudinal study investigating quality of life and nutritional outcomes in advanced cancer patients receiving home parenteral nutrition

**DOI:** 10.1186/1471-2407-14-593

**Published:** 2014-08-15

**Authors:** Pankaj G Vashi, Sadie Dahlk, Brenten Popiel, Carolyn A Lammersfeld, Carol Ireton-Jones, Digant Gupta

**Affiliations:** Cancer Treatment Centers of America® (CTCA) at Midwestern Regional Medical Center, 2520 Elisha Avenue, Zion, IL 60099 USA

**Keywords:** Home parenteral nutrition, Quality of life, Nutritional status, Advanced cancer

## Abstract

**Background:**

In cancer patients where gastrointestinal function is marginal and malnutrition significant enough to result in the requirement for intensive nutrition support, parenteral nutrition (PN) is indicated. This longitudinal study examined the quality of life (QoL) and nutritional outcomes in advanced cancer patients receiving home PN (HPN).

**Methods:**

Fifty-two adult cancer patients (21 males, 31 females, average age 53 years) treated at a specialized cancer facility between April 2009 and November 2011 met criteria. QoL and nutritional status were measured at baseline and every month while on HPN using EORTC-QLQ-C30, Karnofsky Performance Status (KPS), and Subjective Global Assessment (SGA). Repeated measures ANOVA and Generalized Estimating Equations (GEE) were used to evaluate longitudinal changes in QoL and SGA.

**Results:**

Cancer diagnoses included pancreatic (n = 14), colorectal (n = 11), ovarian (n = 6), appendix (n = 5), stomach (n = 4) and others (n = 12). Average weight loss 6-months prior to HPN was 13.2 kg (16.9%). Average weight at initiation of HPN was 62.2 kg. In patients with available follow-up data after 1 month (n = 39), there was a significant improvement in SGA, weight (61.5 to 63.1 kg; p = 0.03) and KPS (61.6 to 67.3; p = 0.01) from baseline. Similarly, after 2 months (n = 22), there was an improvement in global QoL (37.1 to 49.2; p = 0.02), SGA, weight (57.6 to 60 kg; p = 0.04) and KPS (63.2 to 73.2; p = 0.01) from baseline. Finally, after 3 months (n = 15), there was an improvement in global QoL (30.6 to 54.4; p = 0.02), SGA, weight (61.1 to 65.9 kg; p = 0.04) and KPS (64.0 to 78.7; p = 0.002) from baseline. Upon GEE analysis, every 1 month of HPN was associated with an increase of 6.3 points in global QoL (p<0.001), 1.3 kg in weight (p = 0.009) and 5.8 points in KPS (p<0.001).

**Conclusions:**

HPN is associated with an improvement in QoL, KPS and nutritional status in advanced cancer patients, irrespective of their tumor type, who have compromised enteral intake and malnutrition. The greatest benefit was seen in patients with 3 months of HPN, although patients receiving HPN for 1 or 2 months also demonstrated significant improvements.

## Background

Malnutrition is observed in up to 80% of patients with advanced cancer [1–5]. Decreased dietary intake, cancer cachexia (characterized mainly by weight loss and muscle wasting), and nutrition impact symptoms may all contribute to cancer-related malnutrition [6]. Additionally, the treatment modalities involving combinations of chemotherapeutic, radiotherapeutic and surgical regimens are known to produce various acute and chronic symptoms that limit eating and, thereby, exert a profound impact on nutritional status [7, 8]. As a result, identifying and treating malnutrition early in the course of advanced cancer is critical to achieving favorable patient outcomes.

However, when malnutrition is significant enough to result in the requirement for intensive nutrition support in patients with marginal gastrointestinal (GI) function, parenteral nutrition (PN) is indicated. Advanced cancer patients with poor GI function and inadequate or inability to take oral or enteral nutrition may receive PN with the goals of improving QoL as well as nutritionally supporting them while they receive anti-cancer therapy, which may also have a positive impact on survival [9, 10]. The success of PN depends on patient compliance, the intensive consulting and support of all patients and their relatives by a professional and committed nutritionist, and the constructive cooperation between the patient, the nutritionist, the supervising physician and the home care provider [9]. As patients receive their treatments outside the hospital, PN may be continued at home (HPN) as an important adjuvant therapy.

While the administration of HPN has been shown to improve survival in advanced cancer patients [10–13], relatively little is known about the potential quality of life (QoL) benefits of HPN [9, 14, 15]. Of particular mention is the study by Bozzetti et al. who evaluated QoL using the Rotterdam Symptom Checklist questionnaire in advanced cancer patients on HPN and concluded that patients who survive for longer than 3 months have time to benefit in terms of QoL. They further suggested that HPN should only be administered to patients with a Karnofsky Performance Status (KPS) score greater than 50 [16]. While the study by Bozzetti et al. provides useful insights on the QoL benefits of HPN, no studies have systematically documented monthly changes in QoL, nutritional and functional status in advanced cancer patients while receiving HPN.

In this study, we longitudinally investigated the QoL, nutritional and functional outcomes of advanced cancer patients receiving HPN managed by a specialized oncology cancer center.

## Methods

### Study population

This was a longitudinal non-randomized clinical study of 52 adult cancer patients treated at Cancer Treatment Centers of America® (CTCA) located in Zion, IL, Philadelphia, PA and Tulsa, OK between April 2009 and November 2011 who were followed until March 2014. The inclusion criteria for participation in this study were a histologically confirmed diagnosis of cancer, anticipated survival of >90 days, no HPN therapy prior to hospital admission, and no associated liver and kidney problems. All patients had significant cancer cachexia with tumor burden involving multiple organs and compromised GI function such that PN was the only option for fulfilling their protein and caloric needs. Patients who did not give informed consent or refused to participate were excluded from the study. All patients were assured that refusal to participate would not affect their future care in any way.

Patients who chose to participate underwent QoL and nutritional status assessments at baseline and every month while on HPN. All patients underwent active chemotherapy, radiation, or hormonal therapy while enrolled in the study. The treatment regimens were decided by the oncologist of the patients based on the National Comprehensive Cancer Network guidelines when available. All patients received HPN from one national home infusion provider for consistency in therapy delivery and management. In addition, Registered Dietitians (RD) from the home infusion provider communicated monthly with patients, while at home, to obtain study data. Consistent training and education was completed at each participating CTCA site and serving branch of the home infusion provider. This study was approved by the Institutional Review Board at CTCA.

### Home parenteral nutrition

Patients who were candidates for HPN were identified by the Nutrition and Metabolic Support Team (NMST). All patients in the study received a minimum of one month of HPN from one national home infusion provider. PN was indicated following the American Society of Parenteral and Enteral Nutrition (ASPEN) guidelines in that each patient satisfied one or more of the following: compromised GI function, enteral feeding not an option, poor oral intake and moderately or severely malnourished status [17]. All patients were given a daily cyclic infusion. Patients’ calorie and protein needs were estimated using the ASPEN guidelines for critical care. Calorie needs were estimated using 25–30 kcal/kg for BMI <30 and 22–25 kcal/kg of ideal body weight if BMI > =30. Protein needs were estimated using 1.5 to 2 g/kg for BMI < 30 and 2 to 2.5 g/kg of ideal body weight if BMI > =30. If patients had oral intake, the RD estimated calorie and protein obtained and subtracted that from total needs to determine the amount of calories and protein to be provided by HPN. Patients received a Total Nutrient Admixture solution. Calories from lipids were limited to < 30% of total daily requirement. Amino acids were added to meet estimated protein needs, and the remaining calories were provided by dextrose. Most patients received Multivitamin Infusion-13 with Vitamin K, unless contraindicated, and Multi-trace 5, which includes selenium, zinc, copper, manganese and chromium. Patients and at least one caregiver were trained by a home health nurse on how to infuse the HPN. After training, patients or their caregiver infused the HPN. The home infusion provider monitored compliance and response to therapy, and provided a weekly clinical summary to the NMST. Inventory of HPN bags and supplies was also conducted by the home infusion provider and shared with the NMST. Patients were monitored by NMST closely for their nutritional status and recovery of GI function. Adjustments were made to the macronutrient composition of the HPN if oral intake changed significantly. HPN was stopped if therapy goals were reached or the patient no longer qualified for continuation of HPN due to metabolic complication or change in the clinical condition including stopping all aggressive therapies. If patients were able to consume approximately 65% of their estimated calorie needs by mouth or enteral route, they were weaned from HPN.

### Quality of Life and Functional Status Assessment

QoL was assessed at baseline and every month while on HPN using the European Organization for the Research and Treatment of Cancer Quality of Life Questionnaire (EORTC-QLQ-C30). The EORTC QLQ-C30 is a 30-item cancer-specific questionnaire that incorporates 5 functioning scales (physical, role, cognition, emotional, and social), 8 symptom scales (fatigue, pain, nausea/vomiting, dyspnea, insomnia, loss of appetite, constipation, diarrhea), financial well-being scale and a global scale (based on 2 items: “How would you rate your overall health during the past week?” and “How would you rate your overall quality of life during the past week?”). The raw scores are linearly transformed to give standard scores in the range of 0 to 100 for each of the functioning and symptom scales. Higher scores in the global and functioning scales and lower scores in the symptom scales indicate better QoL. A difference of 5 to 10 points in the scores represents a small change, 10 to 20 points a moderate change, and greater than 20 points a large clinically significant change from the patient’s perspective [18]. This instrument has been extensively tested for reliability and validity [19–21]. Functional status was measured using KPS and was determined by the managing clinician. KPS was measured on a scale of 0 to 100 where 0 is dead and 100 is normal health with no evidence of disease.

### Nutritional assessment

All patients in the study were evaluated by an RD. Subjective Global Assessment (SGA) was used to assess nutritional status. The SGA is a clinical technique that combines data from subjective and objective aspects of medical history (weight change, dietary intake change, gastrointestinal symptoms, and changes in functional capacity) and physical examination (loss of subcutaneous fat, muscle wasting, ankle or sacral edema and ascites). After evaluation, patients are categorized into three distinct classes of nutritional status; well nourished (SGA-A), moderately malnourished (SGA-B) and severely malnourished (SGA-C) as described by Detsky et al. [22]. The SGA has been validated in a number of diverse patient populations, including cancer patients [23–27]. It has also been correlated with a number of objective nutritional assessment indicators, morbidity, mortality, and QoL measures [1, 28–33]. At the subjects’ first visit, measurement of height and weight were performed. The subjects wore light clothing and no shoes. Weight and serum albumin were also measured at baseline and every month while on HPN.

### Statistical analysis

The primary outcome of interest was QoL (EORTC-QLQ-C30). The secondary outcomes of interest were nutritional status (SGA, serum albumin and weight), performance status (KPS) and survival. Duration of HPN was calculated as the number of days the patient remained on HPN starting from the date of HPN start. A comparison of baseline characteristics was made between patients with at least 3 months of follow-up data and those with less than 3 months of follow-up data using 2-sample t test or chi-square test depending upon the underlying distribution of the variables.

As part of the initial longitudinal analyses, QoL, nutritional status and performance status were analyzed in three different groups of patients (those with at least 1, 2, or 3 months of follow-up data) using one-way repeated measures ANOVA with polynomial contrasts. Mauchly’s test of sphericity (compound symmetry) was evaluated and if found to be violated, Greenhouse-Geisser epsilon adjustment was employed. Generalized Estimating Equations (GEE) were then used to analyze the longitudinal changes in 3 dependent variables: QoL (as measured using EORTC-QLQ-C30), performance status (as measured using KPS) and nutritional status (as measured using weight and serum albumin). SGA being a categorical variable was not included as a dependent variable in the longitudinal GEE analyses. The GEE procedure extends the generalized linear model to allow for analysis of repeated measurements [34]. GEE analyses do not require a balanced design (i.e., observations at all measurements for each participant), and they accommodate correlated errors due to repeated measures [35]. Unlike repeated measures ANOVA, GEE does not discard all results on a subject with even a single repeated measurement. In addition, GEE allows for a wide variety of correlation structures to be explicitly modeled and is more interpretable that repeated measures ANOVA. Overall survival was defined as the time interval between the date of HPN start and the date of patient’s death from any cause or the date of last contact/last known to be alive. The overall survival was calculated using the Kaplan-Meier method. Log rank test for used to evaluate the equality of survival distribution across groups.

All data were analyzed using IBM SPSS version 20.0 (IBM, Armonk, NY, USA). All analyses were two-tailed, and a difference was considered to be statistically significant if the p value was less than or equal to 0.05.

## Results

### Patient characteristics

Table 1 describes the baseline characteristics of our patient cohort. Tables 2 and 3 compare the baseline characteristics and QoL and nutritional scores respectively of HPN patients available for 3 months or more versus those with less than 3 months of follow-up. Thirty-seven patients had less than 3 months of follow-up while 15 had 3 or more months of follow-up. Table 2 displays no significant differences in the baseline characteristics between the 2 groups. Similarly, Table 3 demonstrates no significant differences in QoL and nutritional status at baseline between the two patient populations. However, there was a significantly greater PN kcal (p = 0.04) and PN protein (p = 0.03) intake in patients with less than 3 months of follow-up compared to those with 3 or more months of follow-up. This finding is not surprising because a majority of patients with less than 3 months of follow-up had expired within 3 months and their mode of nutrition was primarily parenteral. On the other hand, patients with 3 or more months of follow-up were gradually moved from parenteral to enteral nutrition as their condition improved. Finally, there was no significant difference in the actual enteral intake between the two groups.

**Table 1:** **Baseline patient characteristics (N = 52)**

Characteristic	Categories	Number (%)
Gender	•Males	21 (40.4)
	•Females	31 (59.6)
Class of Case	•Analytic	24 (46.2)
	•Non-analytic	28 (53.8)
Cancer Site	•Pancreas	14 (26.9)
	•Colorectal	11 (21.2)
	•Ovarian	6 (11.5)
	•Appendix	5 (9.6)
	•Stomach	4 (7.7)
	•Others	12 (23.1)
Stage at Diagnosis	•1	3 (5.8)
	•2	11 (21.2)
	•3	12 (23.1)
	•4	21 (40.4)
	•Unknown	5 (9.6)
SGA	•Moderately Malnourished	19 (36.5)
	•Severely Malnourished	33 (63.5)
**Characteristic**	**Mean (standard deviation)**	
Age at HPN Start (years)	53.2 (9.4)	
HPN duration (months)	3.4 (2.5)	
Actual weight (Kg)	62.2 (14.6)	
Percent weight loss 6-months prior to HPN start (percent)	16.9 (9.3)	
Albumin (g/DL)	2.9 (0.62)	
Karnofsky Performance Status (KPS)	60.1 (10.8)

**Table 2:** **Comparison of baseline characteristics of HPN patients available for 3-month follow-up versus those available for less than 3-month follow-up**

Characteristic	Categories	Patients with less than 3 months of follow-up (N = 37)	Patients with 3 or months of follow-up (N = 15)	P
Gender	•Males	15 (40.5)	6 (40.0)	0.97
	•Females	22 (59.5)	9 (60.0)	
Class of Case	•Analytic	17 (45.9)	7 (46.7)	0.96
	•Non-analytic	20 (54.1)	8 (53.3)	
Cancer Site	•Pancreas	9 (24.3)	5 (33.3)	0.29
	•Colorectal	9 (24.3)	2 (13.3)	
	•Ovarian	4 (10.8)	2 (13.3)	
	•Appendix	4 (10.8)	1 (6.7)	
	•Stomach	1 (2.7)	3 (20.0)	
	•Others	10 (27.0)	2 (13.3)	
Stage at Diagnosis	•1	2 (5.9)	1 (7.7)	0.94
	•2	8 (23.5)	3 (23.1)	
	•3	8 (23.5)	4 (30.8)	
	•4	16 (47.1)	5 (38.5)	
SGA	•Moderately Malnourished	15 (40.5)	4 (26.7)	0.35
	•Severely Malnourished	22 (59.5)	11 (73.3)	
Age at HPN Start (years)	•Mean	54.1	51.0	0.30

Values in table are numbers.

Values in parentheses are column percentages.

*P < = 0.05.

2-sample t test or chi-square test used to analyze the data.

**Table 3:** **Comparison of nutritional and QoL scores of HPN patients available for 3-month follow-up versus those available for less than 3-month follow-up**

Characteristic	Patients with less than 3 months of follow-up (N = 37)	Patients with 3 or months of follow-up (N = 15)	P
**Nutritional Status**			
Actual weight (Kg)	62.7 (14.4)	61.1 (15.4)	0.72
Percent weight loss 6-months prior to HPN start (percent)	16.4 (8.7)	18.1 (10.8)	0.55
Albumin (g/DL)	2.9 (0.59)	2.9 (0.69)	0.66
**Performance Status**			
KPS	58.6 (10.5)	64.0 (11.2)	0.11
**QLQ-C30**			
General QoL			
Global	38.1 (23.4)	30.1 (15.9)	0.26
General Function			
Physical	53.7 (23.6)	55.5 (25.6)	0.80
Role	21.2 (19.9)	33.3 (32.7)	0.11
Emotional	58.5 (29.5)	59.4 (21.8)	0.92
Cognitive	64.9 (31.1)	64.4 (28.8)	0.96
Social	36.9 (29.4)	37.8 (33.0)	0.93
General Symptom			
Fatigue	70.9 (22.3)	71.8 (23.3)	0.89
Nausea/Vomiting	56.7 (34.6)	45.5 (31.1)	0.28
Pain	54.5 (35.9)	48.9 (38.0)	0.62
Dyspnea	27.9 (30.9)	31.1 (29.4)	0.73
Insomnia	48.6 (31.0)	51.1 (41.5)	0.81
Appetite Loss	73.0 (28.1)	64.4 (32.0)	0.35
Constipation	34.2 (36.4)	40.0 (42.2)	0.62
Diarrhea	30.6 (33.7)	24.4 (36.6)	0.56
Financial Problems	44.1 (38.5)	53.3 (41.4)	0.50
**Actual PN Intake**			
Kcal (per day)	1468.3 (328.0)	1273.2 (238.4)	0.04*
Protein (grams/day)	81.1 (16.4)	70.0 (14.6)	0.03*
**Actual Enteral intake**			
Kcal (per day)	430.7 (703.0)	581.3 (990.7)	0.54
Protein (grams/day)	13.1 (16.6)	10.3 (10.1)	0.55

Values in table are means.

Values in parentheses are standard deviations.

*P < = 0.05.

QoL scores range from 0 to 100 and have no units.

2-sample t test used to analyze the data.

The reasons for discontinuation of HPN in 37 patients before the end of the 90-day study period were ascertained. They were death or hospice (n = 23), change of home care companies (n = 4), refusal to continue HPN (n = 3), elevated liver function tests (n = 2), advanced to tube feeding (n = 2), sepsis (n = 2) and tolerating adequate oral calorie and protein intake (n = 1). The mean duration of HPN was 3.4 months with a range of 0.4 to 11.7 months. A total of 9 patients received HPN for greater than equal to 9 months. Of these 9 patients, only 1 patient developed a long-term complication of hepatic dysfunction.

### QoL and functional status

Table 4 describes the changes in KPS and QoL during HPN using repeated measures ANOVA. Three different groups of patients were analyzed based on the number of months of available follow-up data. The number of patients available for 1, 2 and 3 months of follow-up were 39, 22, and 15 respectively. After one month of HPN (n = 39) there was a significant improvement in KPS (61.6 to 67.3; p = 0.01), role function (26.4 to 46.7; p = 0.001), fatigue (71 to 59.2; p = 0.03), nausea/vomiting (52.3 to 37; p = 0.03), appetite loss (67.6 to 46.3; p = 0.004) and constipation (37 to 22.2; p = 0.03). After 2 months of HPN (n = 22), there was a significant improvement in KPS (63.2 to 73.2; p = 0.01), global QoL (37.1 to 49.2; p = 0.02), physical function (56.4 to 70.1; p = 0.02), role function (29.5 to 55.3; p = 0.01), emotional function (58.7 to 72.3; p = 0.03), fatigue (73.2 to 50.5; p = 0.002) and appetite loss (68.2 to 33.3; p = 0.001). After 3 months (n = 15), there was a significant improvement in KPS (64.0 to 78.7; p = 0.002), global QoL (30.6 to 54.4; p = 0.02), physical function (55.6 to 72; p = 0.04), role function (33.3 to 58.9; p = 0.03), fatigue (71.8 to 43.7; p = 0.01), appetite loss (64.4 to 33.3; p = 0.02) and constipation (40 to 8.9; p = 0.05). Upon univariate GEE analysis (Table 5), every 1 month of HPN was associated with a significant improvement in KPS by 5.8 points (p < 0.001), global QoL by 6.3 points (p < 0.001), physical QoL by 5.8 points (p = 0.005), role QoL by 12.2 points (p < 0.001), emotional QoL by 4.8 points (p = 0.008), social QoL by 6.2 points (p = 0.04), fatigue by 9.1 points (p < 0.001), nausea/vomiting by 7.1 points (p = 0.005), pain by 6.7 points (p = 0.008), insomnia by 6.5 points (p = 0.02), appetite loss by 13.7 points (p < 0.001) and constipation by 8.8 points (p < 0.001). These improvements remained significant after controlling for age, gender and treatment history.

**Table 4:** **Changes in nutritional and QoL parameters during HPN**

Characteristic	Baseline	Month 1	P	Baseline	Month 1	Month 2	P	Baseline	Month 1	Month 2	Month 3	P
	N = 39			N = 22				N = 15				
**Nutritional Status**												
Weight (Kg)	61.5 (13.4)	63.1 (12.7)	0.03*	57.6 (14.1)	59.9 (13.8)	60.0 (12.4)	0.04*	61.1 (15.4)	63.4 (15.0)	63.6 (12.8)	65.9 (13.6)	0.04*
Albumin (g/DL)	2.8 (0.61)	3.1 (0.65)	0.03*	2.9 (0.66)	3.1 (0.59)	4.3 (5.1)	0.26	2.9 (0.69)	3.1 (0.65)	3.3 (0.44)	3.0 (0.57)	0.28
**Performance Status**												
KPS	61.6 (11.2)	67.3 (10.7)	0.01*	63.2 (9.9)	70.0 (11.1)	73.2 (12.9)	0.01*	64.0 (11.2)	67.3 (11.0)	75.3 (14.6)	78.7 (11.2)	0.002*
**QLQ-C30**												
General QoL												
Global	38.6 (20.6)	46.3 (23.1)	0.98	37.1 (18.1)	51.5 (25.7)	49.2 (21.0)	0.02*	30.6 (15.9)	45.6 (27.1)	47.8 (23.4)	54.4 (24.8)	0.02*
General Function												
Physical	59.6 (20.9)	62.4 (16.0)	0.39	56.4 (22.2)	66.1 (17.4)	70.1 (22.1)	0.02*	55.6 (25.6)	65.8 (20.1)	75.6 (23.2)	72.0 (21.8)	0.04*
Role	26.4 (25.3)	46.7 (33.1)	0.001*	29.5 (29.1)	49.2 (37.3)	55.3 (32.7)	0.01*	33.3 (32.7)	36.7 (36.3)	58.9 (31.1)	58.9 (39.8)	0.03*
Emotional	61.3 (22.8)	64.6 (26.3)	0.50	58.7 (22.9)	72.0 (24.5)	72.3 (25.9)	0.03*	59.4 (21.8)	67.2 (27.0)	67.2 (28.4)	72.8 (24.5)	0.19
Cognitive	65.7 (25.5)	68.1 (30.4)	0.57	66.7 (26.7)	73.5 (30.7)	77.3 (27.0)	0.10	64.4 (28.8)	67.8 (29.8)	78.9 (23.1)	64.4 (29.4)	0.09
Social	37.0 (27.6)	46.3 (29.6)	0.79	41.7 (32.8)	54.5 (30.9)	58.3 (27.1)	0.09	37.8 (33.0)	43.3 (27.3)	55.6 (30.6)	51.1 (34.2)	0.30
General Symptom												
Fatigue	71.0 (20.8)	59.2 (26.8)	0.03*	73.2 (21.6)	54.0 (30.3)	50.5 (26.9)	0.002*	71.8 (23.3)	58.5 (34.2)	45.2 (28.3)	43.7 (30.1)	0.01*
Nausea/	52.3 (29.3)	37.0 (31.9)	0.03*	47.7 (26.7)	43.9 (34.7)	36.4 (32.3)	0.30	45.5 (31.1)	48.9 (34.8)	31.1 (30.1)	34.4 (30.5)	0.20
Vomiting												
Pain	51.4 (35.5)	44.0 (29.6)	0.16	46.2 (37.8)	36.4 (31.1)	28.0 (29.3)	0.06	48.9 (38.0)	35.5 (35.6)	27.8 (31.9)	32.2 (31.5)	0.12
Dyspnea	25.9 (27.7)	19.4 (23.0)	0.21	21.2 (28.2)	16.7 (22.4)	16.7 (26.7)	0.74	31.1 (29.4)	17.8 (21.3)	17.8 (27.8)	20.0 (30.3)	0.41
Insomnia	47.2 (32.2)	45.4 (37.5)	0.77	45.4 (37.9)	40.9 (37.0)	28.8 (27.8)	0.18	51.1 (41.5)	48.9 (37.5)	26.7 (28.7)	33.3 (33.3)	0.09
Appetite Loss	67.6 (29.3)	46.3 (38.4)	0.004*	68.2 (28.1)	39.4 (38.0)	33.3 (39.8)	0.001*	64.4 (32.0)	48.9 (39.6)	31.1 (36.6)	33.3 (37.8)	0.02*
Constipation	37.0 (38.3)	22.2 (31.9)	0.03*	33.3 (38.5)	25.7 (29.0)	24.2 (34.4)	0.43	40.0 (42.2)	33.3 (30.9)	22.2 (32.5)	8.9 (26.6)	0.05*
Diarrhea	33.3 (37.4)	25.9 (31.0)	0.25	31.8 (39.1)	21.2 (26.3)	24.2 (37.3)	0.51	24.4 (36.6)	13.3 (16.9)	24.4 (36.6)	26.7 (40.2)	0.58
Financial	47.2 (39.3)	38.9 (32.4)	0.07	48.5 (42.0)	36.4 (37.0)	39.4 (40.7)	0.14	53.3 (41.4)	40.0 (36.1)	37.8 (39.6)	53.3 (39.4)	0.18
Problems												

Values in table are means.

Values in parentheses are standard deviations.

*P < = 0.05.

QoL scores range from 0 to 100 and have no units.

One-way repeated measures ANOVA used to analyze the data.

**Table 5:** **Longitudinal GEE analyses for QoL, nutritional and functional outcomes**

Dependent Variable	Univariate GEE β	P-Value	Multivariate GEE β	P-Value
	95% CI		95% CI	
**Nutritional Status**				
Weight (Kg)	1.3 (0.32 to 2.2)	0.009*	1.2 (0.27 to 2.2)	**0.01**
Albumin (g/DL)	0.26 (−0.12 to 0.6)	0.18	0.25 (−0.1 to 0.6)	0.17
**Performance Status**				
KPS	5.8 (3.8 to 7.8)	<0.001*	5.9 (4.0 to 7.8)	<0.001*
**QLQ-C30**				
General QoL				
Global	6.3 (2.9 to 9.6)	<0.001*	6.2 (2.7 to 9.6)	<0.001*
General Function				
Physical	5.8 (1.8 to 9.9)	0.005*	5.7 (1.7 to 9.8)	0.006*
Role	12.2 (5.9 to 18.4)	<0.001*	12.1 (6.0 to 18.3)	<0.001*
Emotional	4.8 (1.2 to 8.3)	0.008*	4.7 (1.1 to 8.3)	0.01*
Cognitive	1.2 (−2.5 to 4.8)	0.53	1.1 (−2.5 to 4.8)	0.54
Social	6.2 (0.2 to 12.3)	0.04*	6.2 (0.1 to 12.2)	0.04*
General Symptom				
Fatigue	−9.1 (−13.9 to −4.4)	<0.001*	−9.0 (−13.8 to −4.2)	<0.001*
Nausea/ Vomiting	−7.1 (−12.0 to −2.2)	0.005*	−7.0 (−11.6 to −2.5)	0.002*
Pain	−6.7 (−11.7 to −1.8)	0.008*	−6.7 (−11.7 to −1.7)	0.009*
Dyspnea	−3.7 (−9.6 to 2.2)	0.22	−3.6 (−9.6 to 2.3)	0.23
Insomnia	−6.5 (−12.2 to −0.9)	0.02*	−6.4 (−12.1 to −0.8)	0.02*
Appetite Loss	−13.7 (−19.2 to −8.1)	<0.001*	−13.4 (−18.8 to −8.1)	<0.001*
Constipation	−8.8 (−13.8 to −3.7)	0.001*	−8.7 (−13.6 to −3.7)	0.001*
Diarrhea	−0.87 (−8.0 to 6.2)	0.81	−0.6 (−7.5 to 6.3)	0.86
Financial Problems	−0.2 (−4.8 to 4.8)	0.99	−0.09 (−4.9 to 4.7)	0.97

GEE β estimates in the table above reflect changes in the dependent variable for every 1 month increase in time.

Multivariate β estimates were adjusted for age, gender and treatment history.

*P < =0.05.

QoL scores range from 0 to 100 and have no units.

Longitudinal Generalized Estimating Equations used to analyze the data.

### Nutritional status

At baseline (n = 52), 19 (36.5%) patients were SGA-B and 33 (63.5%) were SGA-C. After 1 month of HPN (n = 39), 2 (5.1%) were SGA-A, 20 (51.3%) were SGA-B and 17 (43.6%) were SGA-C. After 2 months of HPN (n = 22), 3 (13.6%) were SGA-A, 13 (59.1%) were SGA-B and 6 (27.3%) were SGA-C. After 3 months of HPN (n = 15), 2 (13.3%) were SGA-A, 12 (80%) were SGA-B and 1 (6.7%) was SGA-C. The improvements in SGA were statistically significant (p < 0.05) at all time points. Table 4 describes the changes in other nutritional parameters (weight and albumin) during HPN using repeated measures ANOVA. After one month of HPN (n = 39) there was a significant improvement in weight (61.5 to 63.1 kg; p = 0.03) and albumin (2.8 to 3.1 g/dl; p = 0.03). After 2 months of HPN (n = 22), there was a significant improvement in weight (57.6 to 60 kg; p = 0.04). After 3 months (n = 15), there was a significant improvement in weight (61.1 to 65.9 kg; p = 0.04). Upon univariate GEE analysis (Table 5), every 1 month of HPN was associated with a significant improvement in weight by 1.3 kg (p = 0.009). These improvements remained significant after controlling for age, gender and treatment history.

### Survival analysis

At the time of this analysis (March 2014), 48 (92.3%) patients had expired. On Kaplan-Meier analysis, median overall survival for the entire patient cohort was 5.1 months (95% CI: 2.8-7.3 months). The median survival for “KPS < =50” patients and “KPS > 50” patients was 6.4 and 4.6 months respectively, log-rank p = 0.25. The median survival for “SGA-B” patients and “SGA-C” patients was 3.2 and 6.5 months respectively, log-rank p = 0.39. Twenty-seven patients survived greater than 6 months while 25 survived less than 6 months. There were no statistically significant differences in the characteristics of those 2 groups although patients surviving less than 6 months had (as expected) more advanced disease at diagnosis. Twelve patients survived greater than 1 year, and of those 12, 5 patients survived greater than 2 years. Of 5 patients surviving greater than 2 years (mean age 53.2 years), 3 are still alive; 2 had colorectal, 2 pancreatic and 1 ovarian cancer; 3 received 3 or more months of HPN; 3 had baseline KPS > 50; 3 were baseline SGA-C; 3 were females; and 3 were previously treated before coming to our institution.

## Discussion

To the best of our knowledge this is the first US-based study to investigate QoL, nutritional status and functional outcomes of advanced cancer patients receiving HPN. We chose the EORTC QLQ-C30 as a valid and reliable tool to assess patients’ QoL. The EORTC QLQ-C30 concentrates on patients’ ability to fulfill the activities of daily life. Clinical practitioners and investigators need to know what happens to a patients’ capacity to fulfill the activities of daily life at work and at home. Consequently, this instrument has an extensive physical functioning scale coupled with a comprehensive symptom inventory.

The key finding of our study was that HPN is associated with an improvement in QoL, nutritional and functional status in patients with advanced cancer having compromised oral/enteral intake and malnutrition. The greatest benefit in terms of QoL, nutritional and functional status was seen in patients who underwent 3 months of HPN, although patients receiving HPN for 1 or 2 months also demonstrated significant improvements.

Few other studies have reported on QoL in patients with advanced cancer on HPN. A study by Bozzetti investigated the changes of QoL in advanced cancer patients during HPN. The study concluded only patients who survive longer than 3 months have enough time to benefit – though only temporarily – in terms of QoL [16]. The study also suggested that HPN be administered to patients with a KPS >50 provided that the medical indication is valid, that there is a positive assessment of well-being, and that the HPN be continued for a 1 month trial as long as there is no worsening of the patient’s physical state [16]. Our study findings are similar, however, we demonstrated an improved QoL, nutritional and functional status starting after one month of HPN and documented improved nutritional status using SGA, a validated tool measuring nutritional status. In addition, we found that baseline KPS <50 was not associated with a higher risk of death and those patients might benefit from HPN as well. Therefore, we suggest that HPN be administered to patients regardless of their KPS scores.

The results of this study have important implications for both clinical and research practices. They suggest that HPN can be initiated in patients with advanced cancer to improve QoL, nutritional status and functional status. Patient QoL is an extremely important outcome measure for cancer patients. How patients feel, physically and emotionally, while they are fighting cancer can have an enormous effect on their ability to carry out normal daily functions as well as on their interpersonal relationships and their ability to work. Routine QoL measurements should be collected to assess the benefits of HPN therapy. Our data show that after one month of HPN therapy, QoL scores begin to improve and patients begin to suffer less from appetite loss, constipation, nausea/vomiting and fatigue. Role function begins to improve along with SGA, weight and KPS Future research should focus on devising the best management practices for timing of nutritional assessment and intervention in advanced cancer patients and testing their value in controlled clinical studies.

Given that individuals cannot survive without nutrient intake, the ability to perform at a level allowing normal to semi-normal activity could not happen without HPN. This allows oral intake to be for pleasure or comfort (if tolerated) rather than it to be forced and measured. Usual key clinical measures may not indicate progress due to the presence of inflammation and the disease process therefore potentially negating the positive outcomes of HPN. However, a recent study by Culine et al. indicated that not only QoL but weight, serum albumin and nutrition risk improved significantly after one month of HPN [36]. Perhaps more importantly, the family and physicians managing these patients noticed the improvement in well-being.

There are several limitations of this study that require acknowledgement. The study has a relatively small sample size of 52 advanced cancer patients with no formal sample size estimation carried out at the study outset. This is due to the fact that the study was limited to a specific population of cancer patients eligible for HPN therapy. As a result, the generalizability of this study’s findings might be limited. In addition, the study lost some participants after each month of HPN therapy due to death, hospice, tolerating adequate calorie and protein intake and other reasons. Because of a relatively small sample size of 52, it was not possible to comprehensively evaluate the predictors of overall survival. However, survival was not the primary endpoint of the study to begin with. Finally, the study lacks a control group, which makes it difficult to make any definitive conclusions about cause and effect. This study also has several strengths including a longitudinal prospective design, no missing data on any EORTC QLQ-C30 variables for the entire study sample; the use of a valid and reliable QoL and nutritional assessment instrument; and the availability of clinical data in nearly all patients. In addition, this study was done in patients receiving HPN in a typical home environment rather than in a research setting which allows direct application to clinical care.

## Conclusions

HPN is associated with an improvement in QoL, nutritional status and functional in advanced cancer patients, irrespective of their tumor type, who have compromised enteral intake and malnutrition. The greatest benefit was seen in patients with 3 months of HPN, although patients receiving HPN for 1 or 2 months also demonstrated significant improvements. When advanced cancer patients cannot receive adequate nutrients enterally or orally and anti-cancer therapy is continued, HPN is an important component of the care plan.

## Abbreviations

QoL: Quality of life
PN: Parenteral nutrition
GI: Gastrointestinal
HPN: Home parenteral nutrition
KPS: Karnofsky performance status
CTCA: Cancer Treatment Centers of America
RD: Registered dietitian
NMST: Nutrition and metabolic support team
ASPEN: American Society of Parenteral and Enteral Nutrition
Kg: Kilogram
BMI: Body mass index
kcal: Kilocalories
EORTC-QLQ-C30: European Organization for the Research and Treatment of Cancer Quality of Life Questionnaire
SGA: Subjective global assessment
ANOVA: Analysis of variance
GEE: Generalized estimating equations
IBM: International Business Machines
SPSS: Statistical Package for Social Sciences
g/dl: Grams per deciliter.
